# Computational integration and meta-analysis of abandoned cardio-(vascular/renal/metabolic) therapeutics discontinued during clinical trials from 2011 to 2022

**DOI:** 10.3389/fcvm.2023.1033832

**Published:** 2023-02-06

**Authors:** Carisa Zeng, Yoon Seo Lee, Austin Szatrowski, Deniel Mero, Bohdan B. Khomtchouk

**Affiliations:** ^1^The College of the University of Chicago, Chicago, IL, United States; ^2^Dock Therapeutics, Inc., Lewes, DE, United States; ^3^Department of BioHealth Informatics, Luddy School of Informatics, Computing, and Engineering, Indiana University, Indianapolis, IN, United States; ^4^Krannert Cardiovascular Research Center, Indiana University School of Medicine, Indianapolis, IN, United States; ^5^Center for Computational Biology and Bioinformatics, Indiana University School of Medicine, Indianapolis, IN, United States

**Keywords:** cardioinformatics, databases, drug repurposing, computation, bioinformatics

## Abstract

Cardiovascular/renal/metabolic (CVRM) diseases collectively comprise the leading cause of death worldwide and disproportionally affect older demographics and historically underrepresented minority populations. Despite these critical unmet needs, pharmaceutical research and development (R&D) efforts have historically struggled with high drug failure rates, low approval rates, and other challenges. Drug repurposing is one approach to recovering R&D costs and meeting unmet demands in therapeutic markets. While there are multiple approaches to conducting drug repurposing, we recognize the importance of bringing together and consolidating discontinued drug information to help identify prospective repurposing candidates. In this study, we have harmonized and integrated information on all relevant CVRM drug assets from U.S. Securities and Exchange Commission (SEC) filings, clinical trial records, PharmGKB, Open Targets, and other platforms. A list of existing therapeutics discontinued or shelved by pharmaceutical/biotechnology companies in 2011–2022 were manually curated and interpreted for insights using information on each drug’s genetic target, mechanism of action (MOA), clinical indication, and R&D information including highest phase of clinical development, year of discontinuation, previous repurposing attempts (if any), and other actionable metadata. This study also summarizes the profiles of CVRM drugs discontinued within the past decade and identifies the limitations of publicly available information on discontinued drug assets. The constructed database could serve as a tool for identifying candidates for drug repurposing and developing query methods for collecting R&D information.

## Introduction

Historically, the cardiovascular, cardiorenal, and cardiometabolic drug discovery enterprise stagnated relative to oncology and other therapeutic areas due to investor market uncertainty and the unique challenges associated with cardiac biology (1). From 1990 to 2012, the percentage of phase 1 cardiovascular drugs declined from 16 to 5% while phase 3 cardiovascular drugs dropped from 21 to 7% (2). A rapid shift in attention and venture capital investment toward oncology and other therapeutic areas deemed to be lower risk and higher reward followed the highly publicized late-stage clinical trial failures of large pharmaceutical companies operating in the cardiovascular disease space during the early-mid 2000s (3). This, in turn, led to significant research and funding disparities that directly impacted patient care in cardiovascular disease (and its associated cardiorenal-metabolic co-morbidities/complications) for the next decade. However, the last few years have been marked by a series of multiple consecutive research and development (R&D) breakthroughs in this therapeutic sector, many of which have resulted in successful corporate mergers and acquisitions in what is now a burgeoning $250 billion market (4, 5).

Drug repurposing is one possible strategy to alleviating the significant R&D costs associated with disease indications of high clinical unmet need, given the steep increase in R&D costs for new drugs over the years and the relatively low number of approved therapeutics (6, 7). However, drug repurposing faces multiple obstacles such as patent exclusivity, regulation measures, information accessibility, and other challenges (8, 9). Drug development details and relevant data are often either confidential or expensive to obtain, while publicly available information is mostly scattered across different platforms and heterogeneous media in the diverse public domain.

Currently, there are many approaches and ongoing efforts in drug repurposing such as experimental and computational strategies to facilitate identification of existing molecules suitable for new indications and the evaluation of their toxicity and efficacy (7, 9). While consolidating information on chemical properties and data-mining existing therapeutic assets may help clarify new directions for specific repurposing opportunities, we recognize another way of contributing to drug repurposing at a larger scale: by building a database of drugs previously removed from the R&D pipelines of major pharmaceutical and biotechnology companies during the last decade (2011–2022). In general, a drug may be shelved or discontinued for a variety of reasons, including strategic corporate decisions (e.g., company realizes their drug asset is not reaching the right patient population or is unlikely to compete profitably in the evolving competitive landscape of pharmaceutical offerings from other companies), toxicity/efficacy issues, or overall safety concerns based on clinical trial design and patient stratification. However, up to 50% of failures have historically resulted due to efficacy concerns (2).

Drugs with known side-effects can also be considered for repurposing (7), for example, the precedential success of repurposing antiemetic thalidomide for multiple myeloma (10). Such undiscovered or undervalued possibilities for drugs to be repurposed or further modified for clinical development spur many biotech companies to in-license drugs that were previously discontinued due to strategic reasons, given that large pharmaceutical companies have finite budgets for R&D and may elect to discontinue less successful candidates (11). Additionally, human genetic data and computational tools available on public platforms can assist in validating the effects of drug assets, given that 2/3 of FDA-approved drugs in 2021 had supportive human genetic evidence (12).

In general, computational resources can be highly informative and actionable to drug hunters seeking a competitive edge to database-driven decision-making for drug repurposing. For example, monitoring the developmental status of a discontinued drug, providing genetic validation of a specific drug target, or looking at the range of clinical indications available for the same genetic target or mechanism of action (MOA) can be helpful in shortlisting prospective repurposing candidates. Such a resource, compiled into a single consolidated database, can provide a comprehensive overview of therapeutics potentially available for in-licensing from their respective parent organizations. Such is the benefit of constructing an open-source database of discontinued drugs in cardiovascular diseases and its associated renal and metabolic co-morbidities (e.g., chronic kidney disease, type 2 diabetes, non-alcoholic steatohepatitis, etc.). Therefore, we have created a database that integrates information from a variety of biomedical and commercial platforms, including information on drug target, clinical and pathway annotations on the target gene, and other factors.

Our work emphasizes the utility of manually curating and rigorously interpreting a comprehensive and narrowly focused database of discontinued pharmaceuticals relevant to cardiovascular/renal/metabolic (CVRM) disease studies from the past decade of clinical R&D activity. Unlike most manuscripts that focus on successes (drug approvals), our study focuses on failures (drug discontinuations). We foresee this as a beneficial knowledge resource for the broader research community interested in investigating drug repurposing opportunities within CVRM disease phenotypes.

## Materials and methods

Our methods consist of three major parts: (1) identifying a timeframe in which drugs are likely to remain available for repurposing and exempt from potential regulatory constraints, (2) defining a set of contents to be included in the database, and (3) search and curation methods.

### Timeframe

The timeframe of our compiled database spans the years from 2011 to 2022 (inclusive). Patent and regulatory measures are among the major factors that hinder drug repurposing processes (8, 9), which can last at most 10 years (6). The timeframe is determined based on the average timespan from investigational new drug (IND) application to FDA approval (13, 14). This timeframe is important because constructing a database focused specifically on therapeutic assets possibly still protected under the aforementioned guidelines can help drug developers make informative repurposing and licensing decisions.

### Search content

While there are numerous data types that might be helpful, we decided to focus on recording the following publicly available entries pertinent to CVRM diseases:

• Drug name.
• Sponsor company.
• Genetic target [target name (usually a protein) and acronyms, otherwise “N/A” for those that are unclear or involve analog/replacement].
• Clinical indications/disease phenotypes.
• Highest stage of clinical development when the drug was discontinued.
• Year of discontinuation.
• Reason for discontinuation (if provided).
• Primary source of information.
• New indications, new name, the highest stage of repurposing and the company that repurposed the drug.

Clinical trial data is identified as crucial information for drug repurposing (15), but the data can either be found on www.clinicaltrials.gov or be publicly unavailable. Our database constitutes repurposing effort records, but these repurposing efforts are not limited to those that have undergone clinical trials. In order to support genetic validation of drug assets, the target names used in the Open Targets Platform were integrated into our database. In addition, annotations of each genetic target are included to support the genetic validation process:

• Clinical annotation from PharmGKB.
• Pathway annotation from PharmGKB.
• Related-to diseases from PharmGKB.

### Search method

Discontinued drugs with indications relevant to CVRM diseases were searched and curated manually by using the collective domain expertise of our co-authors in SEC filings, clinical records, and other data modalities:

• We began our search by navigating to the “Investor Relations” pages of pharmaceutical corporation websites. From here, we were able to search through current and past fiscal presentations, many of which contained concurrent R&D pipeline information. This allowed us to report information directly from the developer regarding the discontinuation of the listed drugs. Once obtaining this record, we performed keyword searches in www.clinicaltrials.gov to carefully scan ongoing clinical trials being conducted to rule out possible repurposing of each given drug.
• The second method used to identify discontinued drugs was to search by drug target (gene/protein name), rather than by company name. Many of the discontinued drugs targeted the same biological pathway, and by searching for that given pathway, new discontinued drugs were identified. Much like the above method, manual curations of www.clinicaltrials.gov keyword searches were performed to ensure no current studies were being conducted on the drug. Additionally, AdisInsight^1^ was used for links to global data along with relevant timelines following the selected drug.

Discontinued projects were also searched by mining 10K/10Q/20F financial form information from the SEC filing archive using EDGAR full text search. The forms were searched using the names of the pharmaceutical companies. In each form, contents were manually searched and curated primarily using the keywords “discontinue” and “remove.”

The repurposing efforts are searched on:

1) Clinicaltrials.gov using the drug name as the keyword.
2) SEC.gov using the drug name as the keyword under file type 10K, 20F, and 10Q.
3) Google.com using keywords, “<name of drug>” repurposed.

All information that is publicly unavailable based on the search method is marked as “NA.”

## Results

After revising the repurposed status and removing all drugs that are still under development, a list of 92 drugs was compiled. The last update time of the list was 07/15/2022 to report all drugs from 2011 to 2022. The database consists of 20 columns, including the information lists in section “Materials and methods.” An example of the entries is shown in Table 1.

**TABLE 1:** Example of row 77 of the data entries in our manually compiled database.

Compound	LY 2127399 (Tabalumab)
Company	Eli Lilly
Gene target	TNFSF13B
Clinical annotation	NA
Pathway annotation	NA
Related to (disease)	Leukopenia, neutropenia, breast neoplasms
Target	Tumor necrosis factor ligand superfamily member 13B
Target synonym*	
MOA	Tumor necrosis factor ligand superfamily member 13B inhibitor/B cell activating factor inhibitors
Indication	Systemic lupus erythematosus/rheumatoid arthritis/kidney failure
Phase	Phase 3
Year discontinued	2013
Reason of discontinuation*	Efficacy
Link*	LLY-2013.12.31-10K (sec.gov)
Repurposed efforts*	Exocrine pancreatic insufficiency, cystic fibrosis
Repurposed drug name*	Sollpura
Repurposed indication*	Exocrine pancreatic insufficiency, cystic fibrosis
Year*	2017
Company*	Anthera
Phase*	3

The direction of the entries is adjusted vertically that actual entries are aligned horizontally. The target entry may be “N/A” if there is not a specific entry found on publicly available sources. *Some drugs may lack information on these fields. Typically, the reason of discontinuation might not be publicly available, and some drug may not have publicly available records of repurposing.

**FIGURE 1:**
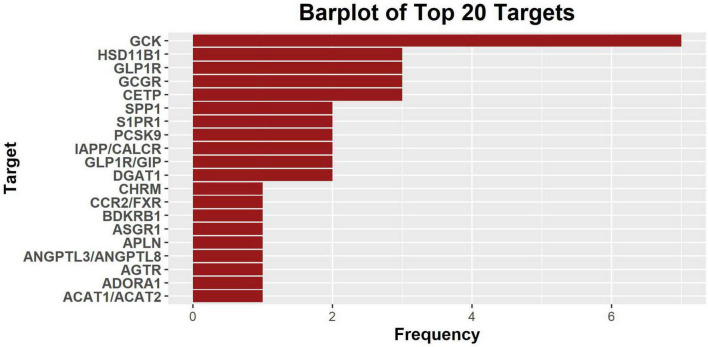
The histogram plot of 20 most common genetic targets of discontinued drugs included in the database. Glucokinase (GCK) is most frequent target among the 51 targets identified, and the corresponding indications are diabetes and renal transplant. Most targets (*n* = 45) only appear once.

The clinical indications, companies, the highest phase of development, and targets have been summarized and plotted using the R programming language. The most common entry for genetic target is “NA,” which means either the target is unclear or not known. There are, in total, 19 assets with “NA” listed as the target and 75 assets with non-“NA” targets. The MOA of “NA”-targeted drugs include: insulin analog, glucagon analog, GLP-1 peptide analog, long-acting basal insulin analog, nitroxyl donor, ghrelin replacements, neurotrophic factor production accelerator, synthetic cyclic peptide and apelin-13 analog (IV), guanosine nucleotide analog (2′-C-methylguanosine), anti-miRNA103/107 oligonucleotide, recombinant human Angiotensin Converting Enzyme (rhACE2), recombinant form of Relaxin 2, mRNA inhibition, and small molecule. Among non-NA target drug assets, there are 56 targets (Supplementary Data) and GCK is the most common target (*n* = 7) (Figure 1). HSD11B1, GCGR, and CETP are the second most common targets (*n* = 3) (Figure 1).

**FIGURE 2:**
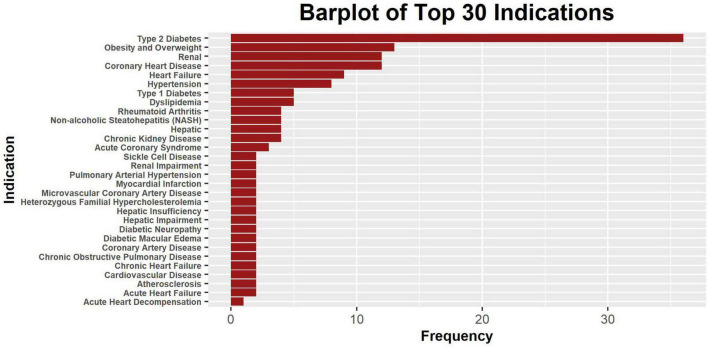
The bar plot of 30 most frequent clinical indications. Only a part of the indications is shown, and some drugs may have multiple indications. The level of specificity of indications varies across drugs, and some indication counts were intended to be redundant to account for differences in specificity.

Meanwhile, most drugs have their clinical indications listed, with type 2 diabetes being the most common (*n* = 37), kidney disease (*n* = 12) and coronary heart disease being the second most common (*n* = 12), and obesity (*n* = 9) as well as heart failure (*n* = 9) being next in ranking (Figure 2). There are in total 94 indications involved in this data set (Supplementary Data).

**FIGURE 3:**
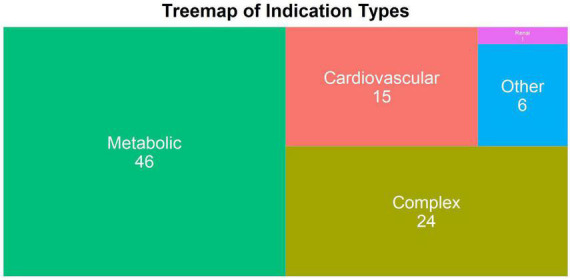
The treemap plot of indication type of all discontinued drugs within the database. The number of indications under a specific indication type is shown on top of each block. The block size is proportional to the number.

The indication types are “cardiovascular,” “renal,” “metabolic,” “complex,” and “other.” Drugs with clinical indications that belong to multiple categories are classified as “complex.” Those with indications outside of the cardiovascular-renal-metabolic (CVRM) categories are considered as “other” (*n* = 5) but involve indications that are related to CVRM in terms of risk factors or comorbidities. Most of the indications are metabolic (*n* = 47) and the second most common is complex (*n* = 25) (Figure 3). It is important to note that there is one drug with indication “renal” (Figure 3), but there are in total 12 renal indications as shown in Figure 2. This is due to how most assets with renal indications are co-developed with other indications, making the listed indication type “complex.”

Five non-CVRM drugs are included in the database: ASK8007 (rheumatoid arthritis), PF-4629991 (rheumatoid arthritis), QAX028 (chronic obstructive pulmonary disease), BMS-986094 (hepatitis C), and AOM1 (non-small lung cancer). While rheumatoid arthritis is an auto-immune disease that is found to be a risk factor for cardiovascular diseases (16), AOM1 is an antibody for SPP1 (osteopontin), the same MOA as ASK8007. QAX028, a muscarinic acetylcholine receptor (CHRM) antagonist, is also included. Although the specific subtype of CHRM that QAX028 targets is not publicly available, the antagonists of CHRM1, CHRM2, and CHRM3 have all been investigated in CVRM-related indications based on documentation from the Open Targets Platform.^2^ Finally, osteopontin (SPP1 or OPN) is a potential target for cardiovascular diseases based on observational data (17).

**FIGURE 4:**
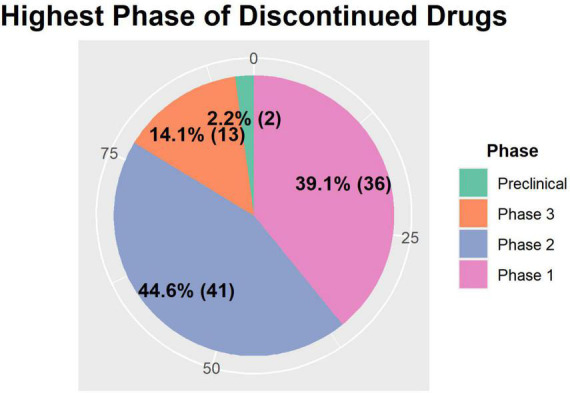
The summary of the highest phase of clinical development before discontinuation. The number and proportion of each phase is displayed in bold, and the numbers (0, 25, 50, and 75) on the outer ring represent the cumulative percentage the area of the pies take up. Preclinical and phase 3 assets take up less than 25% of the assets, whereas phase 2 assets take up 44.7% (*n* = 42) and phase 1 assets take up 39.4% (*n* = 36) of the total assets.

Many of the discontinued drugs reached phase 2 (45.2%) and phase 1 (36.7%), while only two among the 95 assets were discontinued in the preclinical phase (Figure 4). This is contradictory to the expectation that most CVRM drug R&D efforts will terminate at the preclinical phase. The results shown in Figure 4 could be a consequence of the method employed in the data collection process. The major documents searched were quarterly or yearly reports that explicitly mention the discontinuation of projects from the pipeline. The drugs were mostly under clinical trials with some exceptions of successful preclinical projects. Therefore, most drugs that were terminated during preclinical trials were not considered as part of the data to be collected. On the other hand, phase 2 drug assets are generally safe but were discontinued due to lack of efficacy for a specific indication. Out of the 92 assets included in our databases, 52 did not have a reason for discontinuation listed, and are marked as “NA” in our database, while 21 listed reasons either due to efficacy or safety issues for the specific indication. Other commonly cited causes for discontinuation were strategic reasons made by the individual companies. This suggests that these assets could become potential candidates for drug repurposing if more suitable indications are investigated in the future.

**FIGURE 5:**
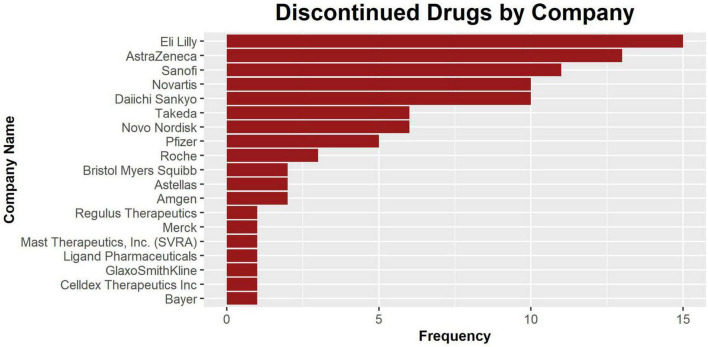
The bar plot of discontinued drugs by company. The companies are listed in order of the number of drug assets recorded in this database. Nine companies have one recorded asset.

There are a total of 20 companies with assets recorded in our database. Among them, Eli Lilly and AstraZeneca have the highest number of discontinued assets (*n* = 15) in the database (Figure 5). While there may be many potential reasons behind why specific pharmaceutical companies have higher numbers of discontinued drugs, frequency in this plot should reflect the amount of effort invested in developing CVRM drugs by a specific company. The full frequency counts are recorded in Table 2.

**TABLE 2:** The frequency table of the pharmaceutical companies with drug assets recorded in the database.

Company	Frequency
Eli Lilly	15
AstraZeneca	13
Sanofi	11
Novartis	10
Daiichi Sankyo	10
Novo Nordisk	6
Takeda	6
Pfizer	5
Roche	3
Amgen	2
Astellas	2
Bristol Myers Squibb	2
Bayer	1
Celldex Therapeutics Inc.	1
GlaxoSmithKline	1
Ligand	1
Mast Therapeutics, Inc. (SVRA)	1
Merck	1
Regulus Therapeutics	1

## Discussion

There are three main highlights to the work presented herein:

1. Many existing public drug databases present incomplete or missing data with limited functional utility for end-to-end evaluation. Our study integrates information from a variety of heterogenous sources using both open-source biomedical and commercial platforms to evaluate previous failures that can facilitate and enable future drug R&D success. Specifically, our study harmonizes and integrates fragmented disparate information on all relevant CVRM drugs (95 drugs in total), including their genetic targets, MOA, disease indications, and highest stage of clinical development (ranging from preclinical to phase 3), along with other actionable information such as discontinuation reasons and any prior repurposing attempts along with the respective trial outcomes.
2. Past drug development studies have often overlooked CVRM drugs as viable repurposing candidates, at least when compared to similar assets in oncology, neurodegeneration, or other disease verticals. Our CVRM-focused database consolidates information on relevant therapeutics across a variety of interrelated cardiovascular disease phenotypes as well as their associated renal and metabolic co-morbidities.
3. Using a large-scale meta-analysis approach, we find that many drugs listed in our database were discontinued in phase 2 clinical trials, suggesting that they are relatively safe in humans but lacked the required efficacy for the tested indications as 17 out of the 92 assets listed efficacy as the reason for discontinuation. This data-driven result challenges previous assumptions that CVRM-related drug assets pose significant safety or toxicity risks indicative of the potential for late-stage phase 3 clinical trial failures (see section “Introduction”). Instead, our database suggests that many of these discontinued drugs may prove to be better suited for other repurposed indications yet pose unremarkable safety risk profiles by themselves. Even for assets discontinued with safety issues, they could be potentially repurposed by developing alternative delivery systems. Therefore, our database can serve as a starting point for evaluating the existing CVRM therapeutics landscape for potential repurposing initiatives that are on-strategy to enterprise R&D pipelines or academic researchers engaged in preclinical investigations.

In general, drug repurposing is crucial for recovering economic and resource allocation loss from unsuccessful drug development projects. Maximizing the potential of success in drug repurposing requires information to be collated from a variety of different sources, but existing information on drug development is mostly fragmented and not easily accessible. Moreover, information on discontinued drugs is seldomly published in peer-reviewed academic literature, and therefore less visible to the broader research community, relative to the more successful therapeutic candidates that have garnered FDA approval and subsequent publication exposure in high-profile clinical journals such as *Lancet* or *New England Journal of Medicine*.

Due to the highly unintegrated and disparate nature of drug development metadata information found in public domain sources, assessing the quality of the entries in this database requires using different platforms in tandem. The four major platforms we used are:

1) SEC.gov for looking up the developmental status and clinical indication of a particular drug. The year and reason for discontinuation may be found here.
2) Clinicaltrials.gov: all the registered clinical trials of a particular drug can be found along with the indications, stage, and sponsor.
3) OpenTargets.org and PharmGKB: the genetic target of a drug and the profile of the target can be found. The profile of a target includes but is not limited to “known drugs,” “safety,” “gene ontology,” and “baseline expression.” For example, GLP, also known as EHMT1, has no data on known drug and safety (see text footnote 2).

Overall, the database allows connection of data from filings to biomedical platforms like Open Targets and PharmGKB. For many drugs, most information including MOA, target, and indication, are left blank on public drug databases. Meanwhile, although Open Targets does indicate whether there are developed drugs for a given target, along with other chemical and biological profiles of the target, it may not display information of developing or discontinued drugs for the target.

Additionally, four current limitations to our database are discussed below:

1) *Timeframe:* Since developmental status of drugs are updated through company filings every quarter and reported on through various media news outlets, some drugs will be later found to be acquired by a new company or eventually become repurposed, divested, or out-licensed for further development. Yet, some drugs seem to be more difficult to repurpose than others and there are several drugs, such as CS1036, that have remained discontinued for more than 10 years. In some cases, a drug may be discontinued and restarted in preclinical studies under the same indication.
2) *Available platform:* There could also be limitations due to potential factual inaccuracies in the publicly available sources and platforms we used for gathering primary source information. Similarly, there is still some missing information on annotations of genetic targets and reasons for discontinuation. The reasons for discontinuation are only provided for a small number of drugs.
3) *Synonyms:* Different synonyms of the same drug or target may yield different search results on the platforms and search engine. Specifically, some drugs are called more by their chemical name in publicly available platforms and would potentially introduce bias in search results if other names are used during a search. In addition, some drugs are synonyms to unrelated items.
4) *Inclusion of non-CVRM drugs:* Although some non-CVRM indications may be relevant to CVRM diseases, and therefore makes it appealing to include non-CVRM drugs to this database, the decision-making process can sometimes become arbitrary and significantly depends on the knowledge of the current community.

These limitations also shed light on the challenges faced by the academic research community. While publicly available information on drug assets is scattered across various platforms, crucial information can remain incomplete or vague. The specific reasons and other contextual information behind halting development is generally missing on top of clinical data often being unavailable. Additionally, other names of drug assets increase the difficulty of tracking their developmental status. Hence, it is important to create a more transparent platform that consolidates pivotal information about failed R&D to facilitate the improvement and repurposing of discontinued therapeutics.

We hope this database will be useful to those looking for consolidated information on drug development insights relevant to cardiovascular, cardiorenal, and cardiometabolic disorders. The entries can be compared based on targets and MOAs, clinical indications, and highest developmental phases. The indications, MOAs, and targets can also be cross compared for inference. For example, diabetes is the most common indication of the drugs in our compiled database, and the target landscape for type I and type II diabetes can be reasonably assessed in comparison to one another. We foresee drug repurposing using our database to be performed by first checking for drug assets that have been discontinued due to efficacy or strategic reasons as well as during highest developmental phases. Then the MOA should also provide a sense of difficulty in repurposing since some targets are more difficult than others to drug. The database can also provide a sense of possible drugs for in-licensing and performance of a particular MOA on a specific or a range of indications.

## Conclusion

Taken together, we have built a database featuring discontinued CVRM-relevant drug assets that have not yet been successfully repurposed and/or have been shelved for strategic reasons from the past decade (2011–2022). We hope our manually curated database and meta-analysis presented in this study will help researchers in the CVRM drug discovery community make more informed data-driven decisions for formulating their own drug repurposing strategies, show potential connections and mechanistic links amongst the various CVRM disease phenotypes, and identify ways of integrating information from multiple disparate heterogenous sources to assist in the divestiture and out-licensing of existing therapeutic assets.

## Data availability statement

The original contributions presented in this study are included in this article/Supplementary material, further inquiries can be directed to the corresponding author.

## Author contributions

All authors listed have made a substantial, direct, and intellectual contribution to the work, and approved it for publication.

## Footnotes

1
https://adisinsight.springer.com/

2
https://www.opentargets.org/
